# Retraction: The Gene Expression Analysis of Blood Reveals *S100A11* and *AQP9* as Potential Biomarkers of Infective Endocarditis

**DOI:** 10.1371/journal.pone.0347627

**Published:** 2026-04-21

**Authors:** The PLOS One Editors

The *PLOS One* Editors retract this article [1,2] due to concerns about compliance with the PLOS Human Subjects Research policy.

The Methods section in [1] reports that the study involved blood samples from patients with clinical suspicion of native valve infective endocarditis and age-matched healthy volunteers, as well as valvular surgical samples from patients undergoing cardiac surgery. Furthermore, the article states that all participants in the study were prospectively enrolled from January 2009 to December 2010 at the Cardiology Department of La Timone Hospital, Marseille, France, and that the study was approved by the Ethics Committee of the Université de la Méditerranée, but it does not report an ethics approval reference number.

Neither the authors nor the institute responded to the journal’s request for additional information and a copy of the ethics approval documentation. In the absence of the requested documentation, PLOS is unable to confirm the article’s compliance with the PLOS Human Subjects Research policy.

All authors either did not respond directly or could not be reached.
